# A case report of pneumonic-type adenocarcinoma diagnosed by transbronchial cryobiopsy after the patient's death

**DOI:** 10.1097/MD.0000000000024296

**Published:** 2021-02-05

**Authors:** Qiquan Zhao, Xiaoli Han, Li Peng

**Affiliations:** a Department of Respiratory and Critical Care Medicine, The People's Hospital of Dazu District, Dazu; b Department of Respiratory and Critical Care Medicine, The First Affiliated Hospital of Chongqing Medical University, Yuzhong District, Chongqing, China.

**Keywords:** pneumonic-type adenocarcinoma, transbronchial cryobiopsy, after the patient's death

## Abstract

**Rationale:**

Due to its nonspecific manifestations, pneumonic-type adenocarcinoma can be easily misdiagnosed as pneumonia, tuberculosis, or other diseases, especially in developing countries where many patients in the early stage refuse invasive examinations. Early recognition of pneumonic-type adenocarcinoma is essential.

**Patient concerns:**

We report a case of pneumonia lung adenocarcinoma diagnosed by frozen lung biopsy after death.

**Diagnoses:**

A 75-year-old male patient was admitted to the hospital on April 24, complaining of 5 months of recurrent coughing, expectoration, and panting, and his symptoms had been worsening over the past month.

**Interventions:**

After obtaining informed consent from the patient's family, transbronchial cryobiopsy was performed at the bedside.

**Outcomes:**

After a positive rescue, the patient died. Pathological examination indicated adenocarcinoma.

**Lessons:**

At present, surgery is still the first choice for the treatment of pneumonic lung cancer, and early diagnosis can remove the tumor as much as possible. Transbronchial cryobiopsy can be used for the collection of pathological samples, especially for the early diagnosis of pneumonic lung cancer.

## 1 Introduction

Pneumonic-type adenocarcinoma (P-ADC) is easily misdiagnosed as an infectious disease in the clinic. It is commonly present in cases of mucinous bronchioloalveolar carcinoma, where tumor cells involve multiple lobes or both lungs. Chest imaging resembles lobar pneumonia. Due to its nonspecific manifestations, P-ADC can be easily misdiagnosed as pneumonia, tuberculosis, or other diseases, especially in developing countries where many patients in the early stage refuse invasive examinations.^[1]^ Because of delays in taking biopsies and making a diagnosis, patients often die quickly after not responding to anti-microorganism treatment. Carcinoma as a cause of pneumonia is reported to affect 0% to 8% in most series.^[2]^ Therefore, early recognition of this type of lung cancer is essential. Here, we report a case of pneumonia lung adenocarcinoma diagnosed by frozen lung biopsy after death. Written informed consent was obtained from the patient for publication of this case report and accompanying images.

## 2 Case presentation

A 75-year-old male patient was admitted to the hospital on April 24, complaining of 5 months of recurrent coughing, expectoration, and panting, and his symptoms had been worsening over the past month. Five months before his admission, he coughed up white frothy sputum and presented to a local community hospital. Considering the diagnosis of pneumonia, he was given empiric treatment with oral anti-bacterial medication but there was no apparent improvement.

**Figure 1:**
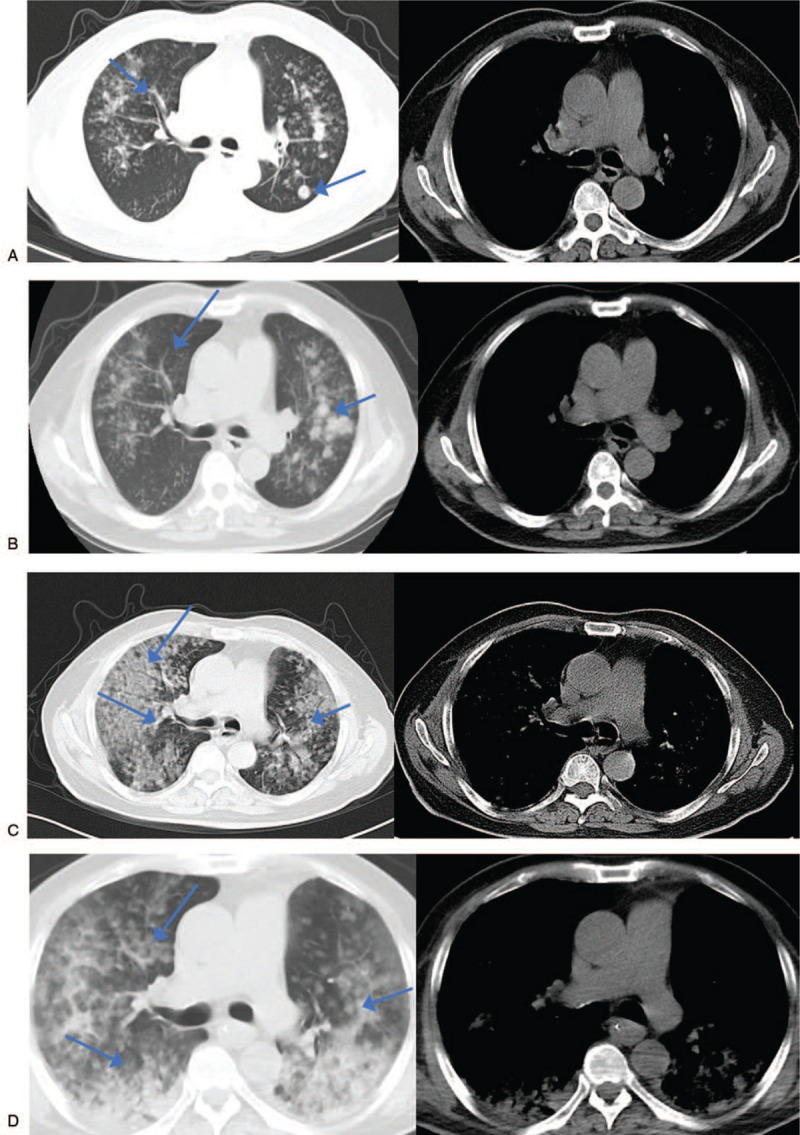
A. January 19, 2020 chest CT indicating a special infectious lesion that needed to be checked and a nodular shadow in the back of the left lower lobe. B. January 26, 2020 chest CT reexamination indicated diffuse lesions of both lungs, scattered ground glass nodes in both lungs and nodular shadow in the posterior segment of the upper lobe of the left lung. The nodular shadow in the back of the left lower lobe had disappeared. C. April 24, 2020 chest CT reexamination indicating diffuse lesions of both lungs and a special pathological bacterial infection lesion that needed to be examined. Compared with the film on January 26, 2020 the foci had increased significantly and additional diagnostic tests were suggested. Scattered ground glass nodes with a withered shape were seen after careful film-reading, which was obviously more severe than the one on January 26. However, the nodular shadow in the posterior segment of the upper lobe of left lung had absorbed slightly. D. May 14, 2020 chest CT reexamination indicating diffuse lesions of both lungs and the special pathological bacterial infection lesion that needed to be examined. Compared with the film on February 24, 2020 the foci in the upper lobe of the right lung was reduced while the one in the lower lobe of both lungs was increased. Scattered ground glass nodes with a withered sign were seen after careful film-reading. CT = computed tomography.

Three months ago, chest computed tomography (CT) showed bilateral diffuse lesions and nodular shadow in his left lower lobe (Fig. 1A. The symptoms were relieved after anti-infection treatment with intravenous piperacillin sulbactam (4.5 g, Q8 h). On January 26, 2020 his chest CT reexamination indicated worsening of the opacity in both lungs (Fig. 1B). Although his doctor suggested he should receive an invasive tracheoscopy examination or biopsy to determine the nature of the disease, the patient refused and was discharged after the improvement of his symptoms.

One month ago, the symptoms such as cough, expectoration, and wheezing worsened again. On April 24, 2020 chest CT reexamination indicated more diffuse lesions of both lungs (Fig. 1C). Upon admission to our hospital on April 24, 2020 the patient was treated with noninvasive ventilation, intravenous piperacillin tazobactam (4.5 g Q8 h), linezolid (600 mg Q12 h), fluconazole (800 mg daily), and methylprednisolone sodium (40 mg Q12 h).

On May 14, 2020 chest CT reexamination indicated diffuse lesions of both lungs, including special infectious lesions that needed to be checked. Compared with the previous film on April 24, 2020 the patient had reduced foci in the upper lobe of the right lung but increased foci in the lower lobes of both lungs (Fig. 1D). On May 17, 2020 since the patient had a worsening condition, tracheal intubation and invasive ventilator-assisted ventilation treatment were applied after communicating with his family. The illness worsened, and at 6:15, May 28, 2020 the patient's heart rate was 60/min, and then he quickly went into cardiac and respiratory arrest. After a positive rescue, the patient died.

If a precise diagnosis is required after a patient dies, the traditional method is to perform an autopsy. Since this causes significant damage to the patient's body and can be distressing to the survivors, the patient's family may refuse, which happened in this case. After a discussion among the whole department, tissue samples were taken from transbronchial cryobiopsy (TBCB) to clarify the diagnosis. After obtaining informed consent from the patient's family, TBCB was performed at the bedside.

**Figure 2:**
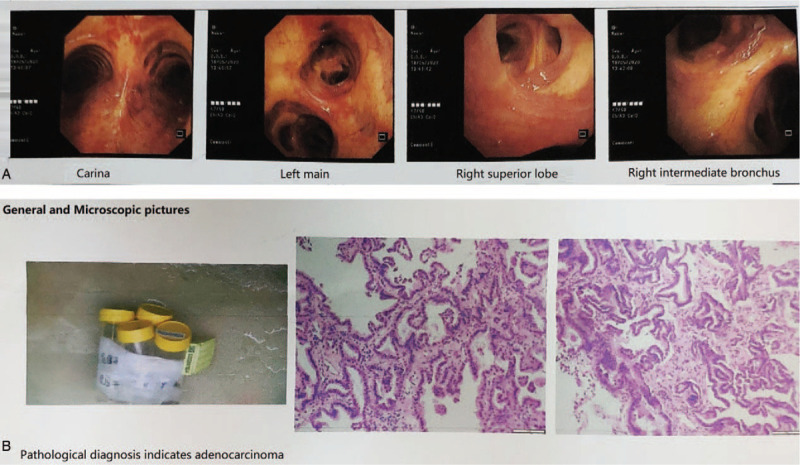
A. The airway was fluent without obvious organisms or stenosis under the scope. B. The results of frozen lung biopsy suggested adenocarcinoma.

The airway was fluent without obvious organisms or stenosis under the scope (Fig. 2A). Four gray-brown soft tissue samples 0.1 cm in diameter were collected at the upper and lower lobes of the left lung, the middle and lower lobes of the right lung. Bacterial and fungal cultures were negative. Pathological examination indicated adenocarcinoma (Fig. 2B).

## 3 Discussion

Pathologically confirmed lung cancer with a chest CT characterized by ground glass opacity or consolidation was defined as P-ADC.^[1]^ It is a special manifestation of peripheral lung cancer. It is a common form of invasive mucinous adenocarcinoma, which is equivalent to bronchiolo-alveolar carcinoma (BAC).^[3,4]^ The early clinical manifestations of pneumonic lung cancer are mild, mainly characterized by cough, expectoration, shortness of breath, no specific symptoms, and imaging is characterized by segmental, lobular, or pulmonary consolidation, similar to pneumonia.^[5]^ With the growth and progression of the cancer cells, it may be complicated with secondary infections, hemoptysis, pulmonary embolism, and other manifestations. CT findings can include honeycomb sign, ground glass sign, nodular sign, withered branch sign, and vascular floating sign that can prompt a diagnosis of pneumonia.^[6,7]^

After anti-infective treatment is ineffective, we need to carefully identify the cause of the “pneumonia.” If a clear diagnosis cannot be made after a routine examination and the effect of anti-infective treatment is poor, especially when the chest CT has honeycomb sign, ground glass sign, nodular sign, withered branch sign, and vascular floating sign, we need to take a tissue biopsy.

The pathological examination of a tissue biopsy is the gold standard for the diagnosis of lung cancer. The traditional sampling methods are biopsy with forceps under bronchoscope, brush biopsy, bronchial lavage, CT-guided lung puncture, and so on. Because pneumonic lung cancer is generally similar to pneumonia, and the cancer tissue is diffusely infiltrative, there is a high risk of percutaneous pneumothorax and bleeding, which limits the application of CT-guided lung biopsy. There are generally no abnormal manifestations such as neoplasms or airway stenosis under fiberoptic bronchoscope, and there are generally no positive results to be seen in the airway. Bronchopulmonary biopsy is the main clinical sampling method.^[5]^ However, bronchopulmonary biopsy has the disadvantage of obtaining small tissue samples.

According to the strict definition of bronchioloalveolar carcinoma according to the WHO classification,^[8–10]^ this carcinoma is an adenocarcinoma growing purely in the form of bronchioles and alveoli, and there is no evidence of interstitial, vascular, or pleural infiltration, so it is usually impossible to make a definitive diagnosis of bronchioloalveolar carcinoma from small biopsy specimens. The final diagnosis must be confirmed after a comprehensive sampling of surgically resected specimens, which greatly reduces the positive rate of diagnosis.

Clinically, there is an urgent need for an effective and minimally invasive early diagnostic technique. Since the 1990s, bronchoscopic cryotherapy has been widely used as an interventional treatment for airway diseases, especially benign and malignant airway stenosis, and has achieved good results.^[11]^ Transbronchial cryobiopsy (TBCB) has been widely used for endotracheal lesions, diffuse lung diseases, and postlung transplantation monitoring because of its minimally invasive nature, high sample quality, low cost, and low risk of complications compared with surgical biopsies. It has been widely used in the United States, Italy, Germany, China, and other countries.^[11–13]^ Another important application of bronchoscopic frozen biopsy is the diagnosis of lung tumors.

The etiological diagnosis of peripheral pulmonary lesions in the past has been a troubling problem for clinicians. Schuhmann et al^[14]^ was the first to compare TBCB with transbronchial forceps biopsy (TBFB) in the diagnosis of peripheral lesions. The results showed that the size of the specimens obtained by TBCB was significantly larger than TBFB the diagnosis rate was significantly higher than that of traditional bronchopulmonary biopsies. It has been confirmed that the application of TBCB in the biopsy of peripheral pulmonary lesions is safe and reliable. The randomized double-blind multicenter trial of Hetzel et al^[15]^ also showed that transbronchial cryobiopsy had a higher positive rate and allowed the identification of molecular targets for adenocarcinoma treatment because of the higher quality of the tissue samples. Schumann et al^[16]^ also found that frozen biopsy had a higher positive rate in the diagnosis of lung cancer, while the risk of bleeding is not higher than that of traditional biopsy. Studies by Arimura et al^[13]^ have shown that transbronchial cryobiopsy can obtain higher quality cancer tissue specimens, which are superior to transbronchial cryobiopsy in gene detection and programmed cell death-Ligand 1 expression detection. Previous research results have therefore proven that transbronchial cryobiopsy is the optimal biopsy method for an early diagnosis of suspected pneumonic lung cancer.

To determine the cause after death, transbronchial cryobiopsy has been performed to confirm the diagnosis of pneumonic lung cancer. After consulting the literature, we found there were no published reports about the use of transbronchial cryobiopsy to make a postmortem diagnosis. This patient had obvious clinical symptoms for about 5 months, but they did not attract much attention. Only ordinary pneumonia was considered as the diagnosis, and although the effect of anti-infective treatment was poor, there were no additional diagnostic tests applied. His condition worsened significantly over his last month, but the delayed diagnosis had missed the best time for treatment, and finally he died because his condition was too severe.

## 4 Conclusion

To sum up, if the clinical effect of anti-infective treatment in patients with suspected pneumonia is not good and the condition progresses, attention must be paid to the differential diagnosis of other diseases, including pneumonic lung cancer, and routine examinations cannot be relied upon to make a clear diagnosis. We need to take tissue biopsies for further differentiation. Transbronchial cryobiopsy can be used for the collection of pathological samples, especially for the early diagnosis of pneumonic lung cancer. An alternative method could even be considered for autopsies of patients who have died of undiagnosed lung disease. At present, surgery is still the first choice for the treatment of pneumonic lung cancer, and early diagnosis can remove the tumor as much as possible. Even if it is not resectable, radiotherapy, chemotherapy, targeted therapy, and immunotherapy can still be considered, which can greatly improve the quality of life of the patients and prolong their survival time.

## Author contributions

**Conceptualization:** Xiaoli Han.

**Data curation:** Xiaoli Han, Li Peng.

**Formal analysis:** Li Peng.

**Funding acquisition:** Xiaoli Han, Li Peng.

**Investigation:** Li Peng.

**Methodology:** Li Peng.

**Project administration:** Li Peng.

**Resources:** Li Peng, Qiquan Zhao and Xiaoli Han.

**Writing – original draft:** Qiquan Zhao.

**Writing – review & editing:** Qiquan Zhao, Li Peng.
